# A novel ganglioside-related risk signature can reveal the distinct immune landscape of neuroblastoma and predict the immunotherapeutic response

**DOI:** 10.3389/fimmu.2022.1061814

**Published:** 2022-12-20

**Authors:** Jiaxing Yang, Lei Han, Yongliang Sha, Yan Jin, Zhongyuan Li, Baocheng Gong, Jie Li, Yun Liu, Yangyang Wang, Qiang Zhao

**Affiliations:** ^1^ Department of Pediatric Oncology, Tianjin Medical University Cancer Institute and Hospital, Tianjin, China; ^2^ National Clinical Research Center for Cancer, Tianjin Medical University Cancer Institute and Hospital, Tianjin, China; ^3^ Key Laboratory of Cancer Prevention and Therapy, Tianjin, China; ^4^ Tianjin’s Clinical Research Center for Cancer, Tianjin, China; ^5^ Department of Cancer Molecular Diagnostics Core, Tianjin Medical University Cancer Institute and Hospital, Tianjin, China

**Keywords:** neuroblastoma, gangliosides, prognosis, immune landscape, dedifferentiation

## Abstract

**Introduction:**

Gangliosides play an essential role in cancer development and progression. However, the involvement of gangliosides in the prognosis and tumor microenvironment (TME) of neuroblastoma is not entirely understood.

**Methods:**

Consensus clustering analysis was performed to identify ganglioside-mediated molecular subtypes. LASSO-Cox analysis was conducted to identify independent prognostic genes, and a novel risk signature was constructed. The risk signature was validated internally and externally. We further explored the independent prognosis value, immune landscape, drug susceptibility, and tumor dedifferentiation of the risk signature. The role of the signature gene B3GALT4 in neuroblastoma was explored *in vitro*.

**Results:**

Seventeen ganglioside-related genes were differentially expressed between INSS stage 4 and other stages, and two ganglioside-related clusters with distinct prognoses were identified. A novel risk signature integrating ten ganglioside-related prognostic genes was established. Across the train set and external validation sets, the risk signature presented high predictive accuracy and discrimination. The risk signature was an independent prognostic factor and constructed a nomogram combining multiple clinical characteristics. In the high-score group, the deficiency in antigen processing and presenting machinery, lack of immune cell infiltration, and escaping NK cells contributed substantially to immune escape. The low-score group was more responsive to immune checkpoint blockade therapy, while the high-score group showed substantial sensitivity to multiple chemotherapeutic drugs. Besides, the risk score was significantly positively correlated with the stemness index and reduced considerably in all-trans retinoic acid-treated neuroblastoma cell lines, indicating high dedifferentiation in the high-score group. Additionally, neuroblastoma cells with downregulation of B3GALT4 present with increased proliferation, invasion, and metastasis abilities *in vitro*.

**Conclusion:**

The novel ganglioside-related risk signature highlights the role of ganglioside in neuroblastoma prognosis and immune landscape and helps optimize chemotherapy and immunotherapy for neuroblastoma.

## 1 Introduction

Neuroblastoma is the most common extracranial solid tumor in children, accounting for only 6-10% of all pediatric malignancies but 12-15% of pediatric cancer-related deaths (1–3). The prognostic heterogeneity of neuroblastoma has been wildly characterized. The 5-year event-free survival (EFS) rate in the low-intermediate risk neuroblastoma exceeds 80%, while the high-risk group, which accounts for half of the total cases, has a 5-year EFS of only 50% (4). Further survival improvement needs more precise prognostic information on neuroblastoma, and novel genetic and molecular predictive biomarkers are urgently required besides known clinical risk factors.

Gangliosides are glycosphingolipids prevalent on the surface of cells, characterized by one or more sialic acid residues on carbohydrate moieties. It is particularly in specialized membrane domains known as lipid rafts and has a role in cell adhesion and signal transduction (5–7). Gangliosides are implicated in cancer development and progression, including tumor proliferation, invasion, angiogenesis, and metastasis (8). Ganglioside GM3 could decrease the phosphorylation of epidermal growth factor receptors and inhibit the proliferation of bladder cancer (9). Ganglioside GM2 is highly expressed in pancreatic ductal adenocarcinoma and correlated with the activation of TGF-β1 signaling and the promotion of tumor invasion (10). Besides, ganglioside GM3 and GD3 are involved in angiogenesis regulation and metastasis in solid tumors (11, 12). However, the role of gangliosides in neuroblastoma is not entirely understood, and the double-edged sword function of gangliosides in regulating malignant characteristics in neuroectodermal-derived malignancies is a critical trait (8, 13, 14). The monosialogangliosides GM3 and GM1 suppress neuroblastoma, glioma, and astrocytoma proliferation by interacting with different growth factor receptors (13). By contrast, GD3 and GD2 of the b-series gangliosides contribute predominantly to tumor-promoting activities in malignancies arising from neuroectodermal cells (13). GD3 is involved in maintaining and enhancing neural stem cell and glioblastoma self-renewal abilities through EGFR activation (15, 16). Besides, GD3 and GD2 promote proliferation, motility, and invasion in various malignancies, including breast cancer, small cell lung cancer, melanoma, and osteosarcoma (13, 17–19). Importantly, GD2 has become one of the most critical tumor markers and immunotherapeutic targets for neuroblastoma. The anti-GD2 monoclonal antibody immunotherapy has been wildly conducted in neuroblastoma clinical management and presents a considerable improvement in high-risk neuroblastoma prognosis. Given the critical and complicated effects of gangliosides on tumors, it is vital to conduct in-depth studies on the role of gangliosides in neuroblastoma to predict prognosis and inform clinical management.

The remarkable efficacy of anti-GD2 monoclonal antibody suggests the tremendous potential of immunotherapy in neuroblastoma. Nonetheless, immunotherapeutic approaches in neuroblastoma continue to face several obstacles. Neuroblastoma has low immunogenicity due to low mutational load and MHC-I expression, resulting in a lack of lymphocyte infiltration and immunological activity in the tumor microenvironment (TME) (20). Additionally, various immune evasion strategies in TME can obstruct lymphocyte infiltration and activation (20). Thus, a comprehensive understanding of the TME is critical for precisely targeting neuroblastoma with immunotherapy. Interestingly, previous studies have shown that gangliosides are involved in regulating TME. Gangliosides on the surface of tumor cells or shed from cells can suppress cytotoxic T cells or dendritic cells, contributing to tumor immune evasion (7, 20). Besides, gangliosides and IFN-γ could synergistically inhibit dendritic cell activity, promoting immune suppression in the TME (21). Therefore, elucidating the role of gangliosides in TME in neuroblastoma could facilitate the understanding of tumor progression and the optimization of immune therapies.

While the involvement of gangliosides in neuroblastoma prognosis and TME remains to be explored, the bioinformatic analysis provides us with a new direction. In the present study, samples in the GSE49710 dataset were clustered based on ganglioside-related gene expression, and a risk signature was constructed to predict neuroblastoma prognosis. Additionally, we investigated the immune landscapes and escape strategies in ganglioside-related risk groups. Immunotherapy response and chemotherapeutic drug sensitivity were further explored in the high-score and low-score groups. Our findings constructed an accurate and effective prognostic signature for neuroblastoma and may help inform the treatment strategy for neuroblastoma.

## 2 Materials and methods

### 2.1 Data acquisition and preprocessing

The workflow of this study was presented in **Figure S1**. Expression data and corresponding clinical information were obtained from the Gene Expression Omnibus (GEO) GSE49710 (n = 498) (22) and ArrayExpress E-MTAB-8248 (n = 223) (23). The Therapeutically Applicable Research to Generate Effective Treatments (TARGET) neuroblastoma gene-expression profile and clinical data (n = 150) were acquired from the UCSC Xena database (http://xena.ucsc.edu/). The GSE49710 cohort was used to construct the risk signature, with the E-MTAB-8248 and TARGET datasets serving as external validation. The clinical baseline characteristics of three data sets were summarized in **Table 1**. Expression data were normalized, and log2 transformed. The expression profile and corresponding immunotherapy information for the GSE78220 cohort were retrieved from the GEO database. The expression data of neuroblastoma cell lines treated with Dimethyl sulfoxide (DMSO) or all-trans retinoic acid (ATRA) was obtained from GSE155000 in the GEO database. Thirty-four genes associated with gangliosides were identified by the Molecular Signatures Database (MSigDB; https://www.gsea-msigdb.org/gsea/msigdb) and previously published literature (**Table S1**) (14).

**Table 1:** Clinical baseline characteristics of three datasets in present study.

Characteristic	GSE49710 (N = 498)	TARGET (N = 150)	E-MTAB-8248 (N = 223)
Gender			
Male	287 (57.6%)	88 (58.7%)	N/A
Female	211(42.4%)	62 (41.3%)	N/A
Stage			
1	121 (24.3%)	0 (0.0%)	29 (13.0%)
2	78 (15.7%)	1 (0.7%)	39 (17.5%)
3	63 (12.7%)	9 (6.0%)	36 (16.1%)
4	183 (36.7%)	120 (80.0%)	89 (39.9%)
4S	53 (10.6%)	20 (13.3%)	30 (13.5%)
Age			
<18 months	305 (61.2%)	32 (21.3%)	104 (46.6%)
≥18 months	193 (38.8%)	118 (78.7%)	119 (53.4%)
MYCN Status			
Normal	401 (80.5%)	119 (79.3%)	176 (78.9%)
Amplified	92 (18.5%)	30 (20.0%)	46 (20.6%)
Unknow	5 (1.0%)	1 (0.7%)	1 (0.5%)
Risk group			
Non–high-risk	322 (64.7%)	31 (20.7%)	133 (59.6%)
High-risk	176 (35.3%)	119 (79.3%)	90 (40.4%)

N/A, not available.

### 2.2 Consensus clustering analysis based ganglioside-related genes

A total of thirty-three ganglioside-related genes were extracted from the GSE49710 dataset. Given the significant difference between International Neuroblastoma Staging System (INSS) stage 4 and other stages (24), seventeen ganglioside-related genes were identified as differentially expressed genes (DEGs) between these two groups. Unsupervised consensus clustering analysis based on ganglioside-related DEGs was performed on GSE49710 and TARGET datasets using the “Consensus Cluster Plus” R package and the “k-means” method to discern diverse expression patterns, with the repetition number set to 1000 to ensure stability (25). The t-distributed stochastic neighbor embedding (t-SNE) analysis was conducted using the “Rtsne” package to investigate the distribution between distinct clusters.

### 2.3 Identification of differentially expressed genes between clusters and functional enrichment analysis

The analysis of differentially expressed genes between clusters was conducted using the “limma” R package according to the specified criteria (|log2FC| ≥1 and adjusted p-value < 0.05). The clusterProfiler R package was used to perform functional enrichment analysis on these DEGs, including Gene Ontology (GO) and Kyoto Encyclopedia of Genes and Genomes (KEGG) analyses (26).

### 2.4 Construction and validation of the ganglioside-related risk signature

Univariate Cox regression analysis using the “survival” R package was performed to screen for potential prognostic genes within ganglioside-related DEGs in the GSE49710 dataset. The least absolute shrinkage and selection operator (LASSO)-penalized Cox regression analysis was then conducted to identify potential signature genes. Finally, the regression coefficients of ten signature genes were determined using multivariate Cox regression. The risk score was calculated by multiplying the expression value of each signature gene by its corresponding regression coefficient.

According to the median risk score, samples were classified as low-score or high-score groups. The scatter dot plots were generated to visualize the association between risk score and survival status. Survival analysis of overall survival (OS) probability was performed to evaluate the risk signature prognostic value using “survival” and “survminer” R packages. Receiver operating characteristic (ROC) curve analysis was performed by the “timeROC” R package to assess the specificity and sensitivity of the risk signature. The unique capability of risk signature was evaluated using principal component analysis (PCA) and the R package “ggplot2.” The ganglioside-related risk signature validation was carried out in GSE49710, TARGET, E-MTAB-8248, and Tianjin cohorts.

### 2.5 Independent prognostic analysis and construction of a nomogram

The “survival” R package was used to conduct univariate and multivariate Cox regression analysis on datasets to determine the risk signature’s predictive significance in the context of recognized prognostic indicators. Furthermore, a nomogram was constructed comprising the risk signature and several established prognostic factors by the “rms” R package to predicate 3-year and 5-year OS. Calibration plots were drawn to assess the accuracy of the nomogram.

### 2.6 Gene set enrichment analysis

Gene Set Enrichment Analysis (GSEA) was conducted to investigate the biological function differences in low-score and high-score groups using the R packages “clusterProfiler” and “enrichplot.” The reference gene set (“c2.cp.kegg.v7.5.1.symbols.gmt”) was acquired from MSigDB (http://www.gsea-msigdb.org/gsea/downloads.jsp).

### 2.7 Immune landscape of the risk signature

The low immunogenicity of neuroblastoma could be attributed to a low mutational burden and impairment in the antigen processing and presenting machinery (APM) (20). APM scores (APS) were derived in the previous study by gene set variation analysis (GSVA) based on 18 APM-related genes to represent antigen processing and presentation efficiency (**Table S2**) (27). As an integral element of APM, MHC-I mediates the recognition and lysis of neuroblastoma cells by cytotoxic T lymphocytes (CTL). To estimate the quantity of MHC-I activity, we obtained the gene set associated with the MHC-I protein complex pathway from the MSigDB (**Table S3**). Subsequently, the enrichment score (ES) was calculated in the single-sample gene set enrichment analysis (ssGSEA) using the “gsva” R package to reflect MHC-I activity, which was defined as the MHC score in this study.

The immune infiltration landscape was investigated by calculating infiltrating scores of 30 different types of TME cells by ssGSEA (**Table S4**) (28, 29). Besides, immune, stromal, and ESTIMATE scores were calculated by the “ESTIMATE” algorithm to reveal the distinct immune microenvironments between risk groups (30).

Immune function-related gene sets, including interferon receptor and natural killer cell cytotoxicity, were gathered from the Immunology Database and Analysis Portal (ImmPort, http://www.immport.org) database (**Table S5**) (31). Immune function activities were measured by ssGSEA and compared between risk groups.

### 2.8 Development of the stemness index

The messenger ribonucleic acid stemness index (mRNAsi) was developed using the one-class logistic regression machine learning algorithm (OCLR) based on pluripotent stem cell samples from the Progenitor Cell Biology Consortium dataset (https://www.synapse.org/, accessed on 16 January 2022). The mRNAsi had been widely used for tumor dedifferentiation and stemness prediction (32–34). The workflow was available on https://bioinformaticsfmrp.github.io/PanCanStem_Web/. The mRNAsi value was normalized to 0-1, with increased mRNAsi indicating a greater degree of dedifferentiation. The stemness index model was constructed in this work, and the mRNAsi was estimated in the GSE49710 dataset. Considering the effect of tumor purity on mRNAsi, we corrected for mRNAsi using tumor purity generated by the ESTIMATE algorithm, and the corrected mRNAsi (c_mRNAsi) was calculated as mRNAsi/tumor purity.

### 2.9 Drug sensitivity analysis

The half-maximal inhibitory concentration (IC50) of commonly used chemotherapeutic agents was predicted by the “pRRophetic” R package to characterize chemosensitivity in high-score and low-score groups (35).

### 2.10 Immunohistochemistry

A total of forty-six paraffin-embedded neuroblastoma specimens were collected at Tianjin Medical University Cancer Institute and Hospital. This study complied with the Declaration of Helsinki and was approved by the Ethics Committee of Tianjin Medical University Cancer Institute and Hospital (E20210027). Sections were deparaffinized with xylene for 30 mins and gradient concentrations of alcohol followed by rehydration. The heat-induced epitope retrieval was conducted by the Tris/EDTA buffer (Solarbio, Beijing, China), pH of 9.0, at 120°C for 3 mins, and the sections were immersed in 3% hydrogen peroxide for 30 min and incubated with the primary ST8SIA2 antibody (dilution 1:100; Rabbit polyclonal, 19736-1-AP; Proteintech), B3GALT4 antibody (dilution 1:100; Rabbit monoclonal, ab169759; abcam), and CD8 antibody (dilution 1:4000; Mouse monoclonal, 66868-1-Ig; Proteintech) at 4°C overnight. After washing with PBS and incubation with the secondary antibody (PV-6001; ZSGB-BIO; Beijing, China) at 37°C for 1 hour, the antigens were detected using DAB chromogen and counterstained with hematoxylin for 1 min. The immunoreactivity score (IRS) was generated for semi-quantitative expression and scored by two independent, experienced pathologists blinded to the clinical information. The inconsistencies were discussed to reach a unified result. The IRS considered staining intensity and the percentage of positive tumor cells. The staining intensity was assessed in four grades, including negative staining (0 points), weak staining (1 point), moderate staining (2 points), and strong staining (3 points). The percentage of positive tumor cells in the section was divided into five grades, including 0-5% (0 point), 6-25% (1 point), 26-50% (2 points), 51-75% (3 points) and 76-100% (4 points). The sample IRS was calculated by multiplying scores of the staining intensity and percentage of positive tumor cells. The precents of CD8^+^ T cells were quantified as the proportion of CD8A-positive cells in all cells on 200× photographs.

### 2.11 Cell lines and cell culture

Neuroblastoma cell lines 9464D and 975A2 were gifted from Dr. Rimas Orentas at Seattle Children’s Research Institute. Neuroblastoma cells were maintained in the high-glucose DMEM medium (Gibco) containing 10% FBS (BI) and 1% penicillin/streptomycin (Gibco). The cells were cultured in a humidified incubator at 37°C in a 5% CO2 atmosphere.

### 2.12 Cell transfection

The small interfering RNA (siRNA) targeting B3GALT4, and negative control siRNA (si-NC) were purchased from General Biol (Anhui, China). The manufacturer’s instruction was followed for cell transfection. Neuroblastoma cells were transfected with siRNA using the transfection reagent Lipofectamine^®^2000 (Invitrogen). The transfected cells were collected for further experiments after 24h. Quantitative realtime PCR assay and western blot analysis were performed to verify the knockdown efficiency.

### 2.13 Western blot analysis

Cells were lysed in RIPA lysis buffer (Solarbio) for protein extraction, and the protein concentrations were evaluated by the BCA method. The proteins were separated by 10% SDS-polyacrylamide gel electrophoresis (PAGE) and transferred to PVDF membranes. After incubation in 5% skimmed milk for 1 h at room temperature, the membranes were incubated overnight at 4°C with primary antibodies against B3GALT4 (dilution 1:1000; Rabbit monoclonal, ab169759; abcam). After incubation with the secondary antibody and wash with TBS-T three times, the band images were visualized by the enhanced chemiluminescence kit.

### 2.14 Real-time quantitative PCR

The total RNA was extracted from cells by Trizol reagent (Invitrogen) and converted to cDNA using the PrimScript RT Master Mix (Takara). cDNA amplification was carried out by SYBR Green PCR Kit (Takara) according to the program: 5 seconds at 95°C for the denaturation, 34 seconds at 60°C for annealing, followed by 30 seconds at 72°C for extension, and forty cycles were completed in total. The primer sequences were designed as follows: B3GALT4: F: 5’-AACGCCATTCGGGCATCTT-3’, R: 5’-GTTGCGGTAGGAATCCTGGAA-3’; GAPDH: F: 5’-ACCCTTAAGAGGGATGCTGC-3’, R: 5’-CCCAATACGGCCAAATCCGT-3’. The 2−ΔΔCt value was employed to quantify the relative gene expression levels with GAPDH as the endogenous control.

### 2.15 Cell proliferation and colony forming assay

Cell viability was measured by the Cell Counting Kit-8 (CCK8) assay. Neuroblastoma cells were plated into 96-well plates for 24h, 48h, and 72h, followed by adding 100µ CCK8 solution (Solarbio) and incubating for 2 hours at 37°C. The absorbance of each well was measured at a wavelength of 450 nm (OD450) with a microplate reader. Cells were planted and cultured for 2 weeks in each well of a 6-well plate for cell colony formation assay. The colonies were fixed with 4% paraformaldehyde for 15 minutes, stained with 0.1% crystal violet for 20 minutes at room temperature, and quantified by ImageJ software after being photographed.

### 2.16 Cell invasion and migration assay

The transwell assay was performed to evaluate cell invasion and migration ability. Eight-micrometer pore-size transwell filters (Corning) were put in a 24-well plate for the migration assay, while the upper chambers plated with matrigel (BD Biosciences) for the invasion assay. Cells in 200 μl FBS-free medium were seeded onto each upper chamber, and the lower chamber was added with 600 μl medium with 10% FBS. After being cultured for 24 hours at 37°C, these invasive and metastatic cells in the lower side of the filter were fixed by 4% paraformaldehyde, stained with 0.1% crystal violet solution, and photographed.

### 2.17 Sample collection for RNA sequencing in the Tianjin cohort

Twenty-six neuroblastoma biopsies and corresponding clinical information were collected at Tianjin Medical University Cancer Institute and Hospital. This study complied with the Declaration of Helsinki and was approved by the Ethics Committee of Tianjin Medical University Cancer Institute and Hospital (E20210027).

### 2.18 RNA quantification and qualification

RNA quantification and qualification were performed according to the following steps. RNA purity and concentration were generated by NanoDrop 2000, and RNA integrity and quantity were quantified by the Agilent 2100/4200 system.

### 2.19 Library construction

The messenger RNA was extracted from total RNA and fragmented into 300-350 bp fragments. The reverse transcription was conducted using fragmented RNA and dNTPs (dATP, dTTP, dCTP, and dGTP) to synthesize the first strand cDNA, followed by the synthesis of the second strand cDNA. After the double-strand cDNA remaining overhangs were converted into blunt ends by exonuclease/polymerase, 3’ ends of DNA fragments were adenylated, and sequencing adaptors were ligated to the cDNA. Subsequently, the library fragments were purified. The PCR was used to amplify the template, and the product was purified to form the final library.

### 2.20 Sequencing and quality control of the raw data

After library preparation and sample pooling, Illumina sequencing was performed on the samples. Raw data in the formation of FASTQ were processed through in-house perl scripts. Clean data were formed by reads without low-quality or adapter and ploy-N. The clean data’s Q20, Q30, and GC content were assessed. The clean reads were mapped to the silva database to eliminate the rRNA.

### 2.21 Reads mapping and quantification of gene expression level

Paired-end clean reads were aligned to the reference genome (hg19) using Hisat2 (36). Featurecount was used to count the reads numbers mapped to each gene (37).

### 2.22 Statistical analysis

All statistical analysis was performed through R software (version 4.1.2). Survival curves were generated by the Kaplan-Meier method and log-rank test for statistical tests. Spearman rank correlation was used to analyze the correlations between continuous variables. The Mann-Whitney Wilcoxon or Kruskal-Wallis test was used to compare continuous variables between groups. The Pearson chi-square test was used to compare categorical variables across groups. Two-sided p < 0.05 was considered statistically significant.

## 3 Results

### 3.1 Consensus clustering analysis of ganglioside-related genes identified two clusters of neuroblastoma with different outcomes

**Figure 1:**
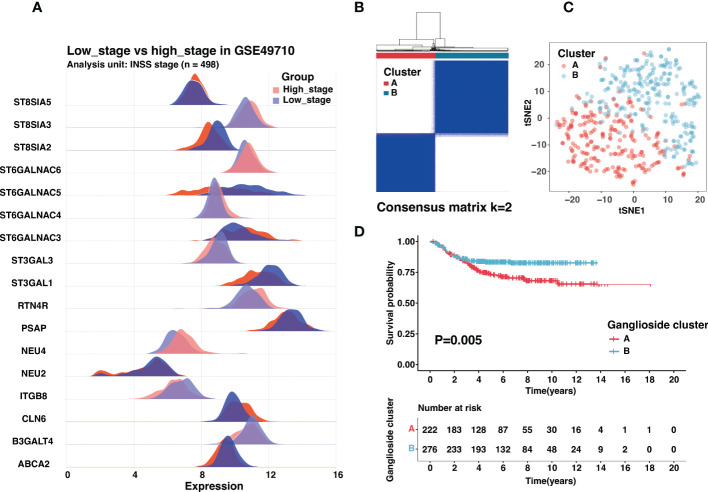
Identification of ganglioside-related clusters in the GSE49710 dataset. **(A)** The expression of ganglioside-related genes that were differentially expressed between samples with high stage (INSS stage 4) and low stage (other stages). **(B)** Identification of two ganglioside-related clusters according to the consensus clustering matrix (k = 2) in the GSE49710 cohort. **(C)** The t-SNE analysis revealed a clear distinction between the two clusters. **(D)** Kaplan–Meier curves of overall survival (OS) in the GSE49710 cohort between different clusters.

The International Neuroblastoma Staging System (INSS) had been wildly used in neuroblastoma clinical management, and INSS stage 4 was an independent risk factor for neuroblastoma (4). Besides prognosis, there were also significant differences in biological characteristics between stage 4 and other stages. To screen crucial ganglioside-related genes in neuroblastoma, we identified seventeen ganglioside-related DEGs between the INSS stage 4 (high stage) and other stages (low stage) (**Figure 1A**). Consensus clustering analysis was performed to classify patients with distinct ganglioside-mediated patterns in GSE49710 based on seventeen ganglioside-related DEGs expression. k = 2 was selected as the ideal option for cluster construction, and 498 samples were allocated to clusters A and B, with 222 and 276 samples, respectively (**Figure 1B**, **Figure S2**; **Table S6**). As shown in **Figure 1C**, the two clusters could be clearly distinguished in the t-SNE analysis. Survival analysis revealed the significant survival advantage of cluster B over cluster A (P=0.005, **Figure 1D**). Additionally, the TARGET dataset was used to verify the consensus clustering result. Two distinct clusters with significantly different prognoses were identified in the TARGET dataset (**Figure S3A-M**; **Table S7**), suggesting the stability of the clustering result.

The analysis of the differentially expressed genes was performed between two clusters in the GSE49710 dataset to explore the further difference in the biological function in identified clusters. One hundred and eight DEGs were finally identified according to |logFC| > 1 and adjusted p-value < 0.05 (**Table S8**). Interestingly, GO functional enrichment analysis showed these DEGs were significantly enriched in neural crest cell development of biological processes, the postsynaptic membrane of cellular components, and signaling receptor activator activity of molecular function (**Figure S4**). In the KEGG functional enrichment analysis, DEGs were enriched in the neuroactive ligand receptor interaction and cAMP signaling pathways (**Figure S4**). These results suggested a potential role for gangliosides in neuroblastoma differentiation. Consistent with the functional enrichment in receptor ligand activity, the role of gangliosides in cancer cell signaling had been widely characterized (18).

### 3.2 Development and validation of the novel ganglioside-related risk signature

**Figure 2:**
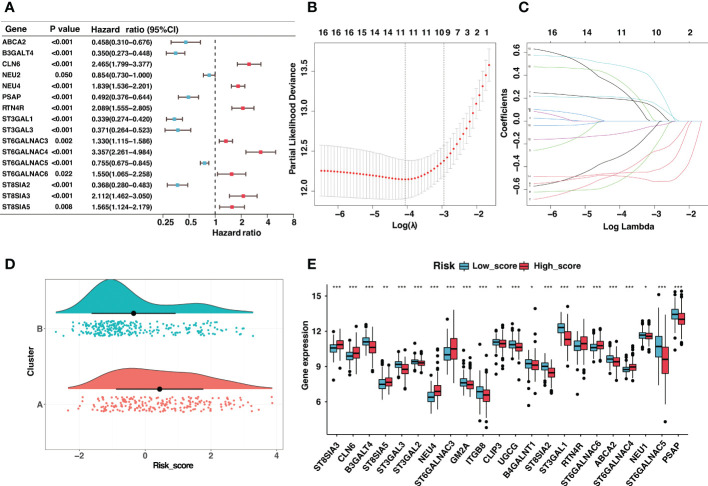
Construction of the ganglioside-related risk signature. **(A)** The forest map of ganglioside-related differentially expressed genes (DEGs) in GSE49710 cohort generated by univariate Cox analysis (P < 0.05). **(B,C)** LASSO Cox regression analysis of ganglioside-related prognostic DEGs. **(D)** The distribution of risk scores between two ganglioside-related clusters. **(E)** The expression of ganglioside-related genes between the low-score and high-score group. (*: P < 0.05, **: P < 0.01, ***: P < 0.001).

?>Given the prognostic significance of different clusters, a ganglioside-related risk signature based on two clusters was developed in the GSE49710 cohort to predict individual prognosis accurately. Firstly, the univariate Cox regression analysis of ganglioside-related DEGs was conducted, and sixteen potential prognostic genes were recognized (**Figure 2A**; **Table S9**). Then, Lasso-penalized Cox regression analysis was performed, followed by multivariate Cox regression to identify ten independent prognostic genes and corresponding regression coefficients. These genes included ABCA2, B3GALT4, NEU4, ST3GAL1, ST3GAL3, ST6GALNAC4, ST6GALNAC5, ST6GALNAC6, ST8SIA2, and ST8SIA3 (**Figures 2B, C**). These ten genes were used to establish the ganglioside-related risk signature, and the risk scores were derived from the expression values of each signature gene and its corresponding regression coefficient. As shown in **Figure 2D**, cluster A presenting with a poor prognosis, received significantly higher risk scores than cluster B. Samples were then divided into high-score and low-score groups based on the median risk score. The expression of ganglioside-related genes was substantially different between the two groups, with eight elevated genes in the high-score group (**Figure 2E**).

**Figure 3:**
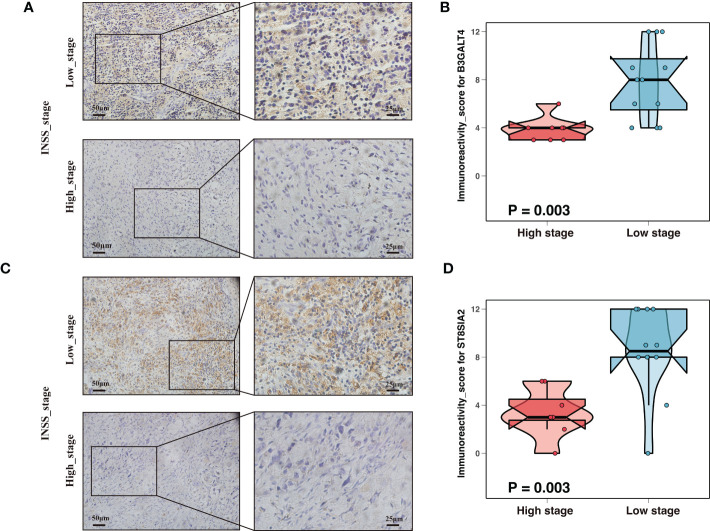
Expression validation of ganglioside-related genes by immunohistochemistry (IHC). **(A)** Representative IHC images showing the expression of B3GALT4 in different stages. Magnification, ×200, ×400. **(B)** The comparison of B3GALT4 immunoreactivity score (IRS) between samples with high stage and low stage. **(C)** Representative IHC images showing the expression of ST8SIA2 in different stages. Magnification, ×200, ×400. **(D)** The ST8SIA2 immunoreactivity score (IRS) comparison between samples with high stage and low stage.

Neuroblastoma with high expression of the b-series gangliosides, including GD1b and GT1b, typically presented an excellent prognosis (14). Interestingly, B3GALT4 catalyzed the first step in converting GD2 to more complex b-series gangliosides. Considering B3GALT4 serves as the connecting link of ganglioside in neuroblastoma, we further performed immunohistochemistry to validate the expression of B3GALT4 in clinical neuroblastoma specimens. Consistent with the results in the GSE49710 dataset, the immunohistochemistry analysis showed a significantly low expression of B3GALT4 in samples with a high stage (**Figures 3A, B**). ST8SIA2 was involved in the developmental regulation of polysialic acid and modulated neuroblastoma adhesion and metastasis. Interestingly, mRNA levels of ST8SIA2 were highest in stages 1 and 4s neuroblastoma (38). Consistent with this result, our study also showed that the expression level of ST8SIA2 was significantly upregulated in low-stage samples (**Figures 3C, D**).

**Figure 4:**
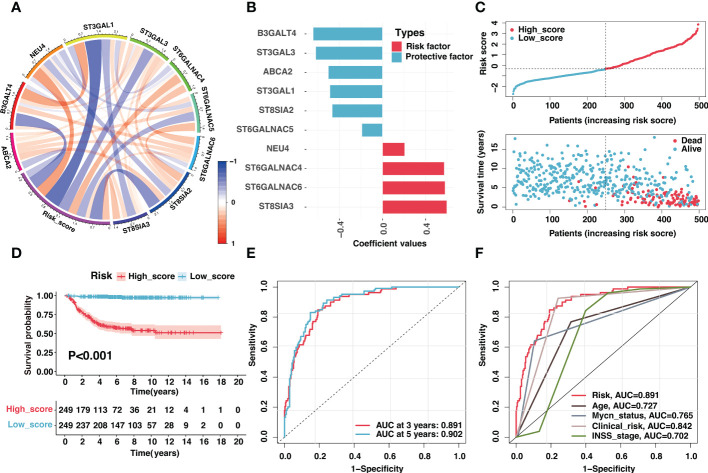
Validation of the ganglioside-related risk signature in the GSE49710 dataset. **(A)** The correlation coefficient between risk score and signature genes. **(B)** Coefficient values of ten signature genes. **(C)** The distribution of the risk score (top) and survival time (bottom) in the GSE49710 cohort. **(D)** Kaplan–Meier curves of overall survival (OS) in GSE49710 cohort between different risk groups. **(E)** The receiver operating characteristic (ROC) curves of the risk signature for 3-year and 5-year OS prediction in the GSE49710 cohort. **(F)** The ROC curves of clinical prognostic factors for 3-year OS prediction in the GSE49710 cohort.

Besides, these signature genes were interconnected (**Figure 4A**), and the corresponding regression coefficients were presented in **Figure 4B** and **Table S10**. The risk signature was validated in the GSE49710, E-MTAB-8248, and TARGET cohorts. Samples in the GSE49710 cohort were divided into two groups based on the median risk score, and an increase in risk score was associated with a decrease in survival time (**Figure 4C**). As expected, those samples classified as the high-score group had a considerably poorer prognosis than samples classified as the low-score group (**Figure 4D**). Additionally, ROC analysis demonstrated that the area under the curve (AUC) values of the risk signature for 3- and 5-year OS prediction were 0.891 and 0.902, respectively (**Figure 4E**). The risk signature performed better in predicting 3-year OS than established clinical prognostic factors (**Figure 4F**).

Moreover, validation was conducted on the E-MTAB-8248 and TARGET cohorts. Consistent with the train set, the increase in risk score was accompanied by a decrease in survival time in E-MTAB-8248 (**Figure S5A**) and TARGET (**Figure S5E**) cohorts. The risk signature could accurately predict 3-year OS in E-MTAB-8248 (**Figure S5B**) and TARGET (**Figure S5F**) cohorts, with AUC values of 0.807 and 0.667, better than clinical characteristics (**Figure S5C** and **S5G**). Importantly, the risk signature had a vital prognosis predictive value in both cohorts, exhibiting significantly poor prognosis in the high-score group (**Figure S5D** and **S5H**). The PCA analysis suggested that the high-score group could also be separated from the low-score group in all cohorts (**Figure S5I-K**). In addition, even in children in COG high-risk group or older than 18 months, the prognosis of samples with a high score was significantly worse than that of low-score samples (**Figures S6A-D**).

**Figure 5:**
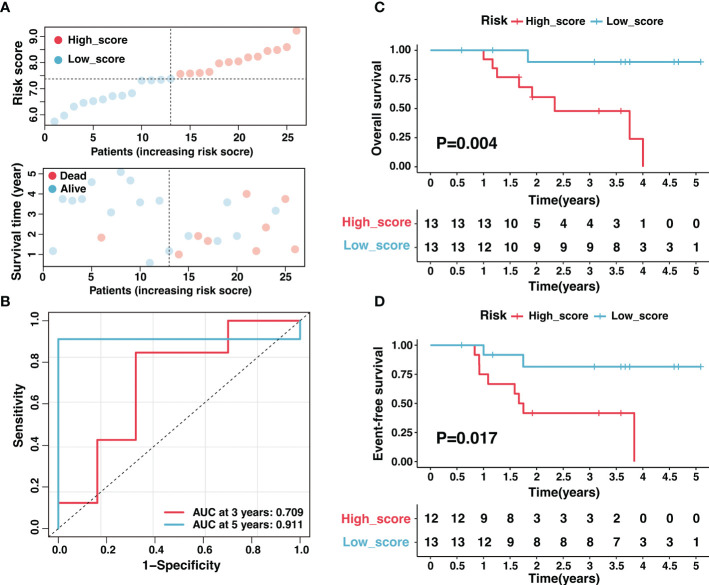
Validation of the ganglioside-related risk signature in the Tianjin cohort. **(A)** The distribution of the risk score (top) and survival time (bottom) in the Tianjin cohort. **(B)** The ROC curves of the risk signature for 3-year and 5-year OS prediction in the Tianjin cohort. **(C, D)** Kaplan–Meier curves of overall survival **(C)** and event-free survival **(D)** in Tianjin cohort between different risk groups.

Additionally, we validated the reliability of risk signature with tissue samples in our center. A total of twenty-six samples with RNA-seq data were included in this study, and the corresponding clinical characteristics were shown in **Tables S11–12**. As expected, high-score samples in the Tianjin cohort presented low survival time (**Figure 5A**). The risk signature performed excellent sensitivity and specificity in predicting 3-year and 5-year OS (**Figure 5B**). The overall survival and event-free survival were significantly worse for the sample in the high-score group compared with low-score samples (P < 0.05, **Figures 5C, D**). In conclusion, the risk signature could effectively predict neuroblastoma prognosis after thorough evaluation and validation.

### 3.3 Clinical correlation analysis, independent prognosis analysis, and construction of a nomogram

**Figure 6:**
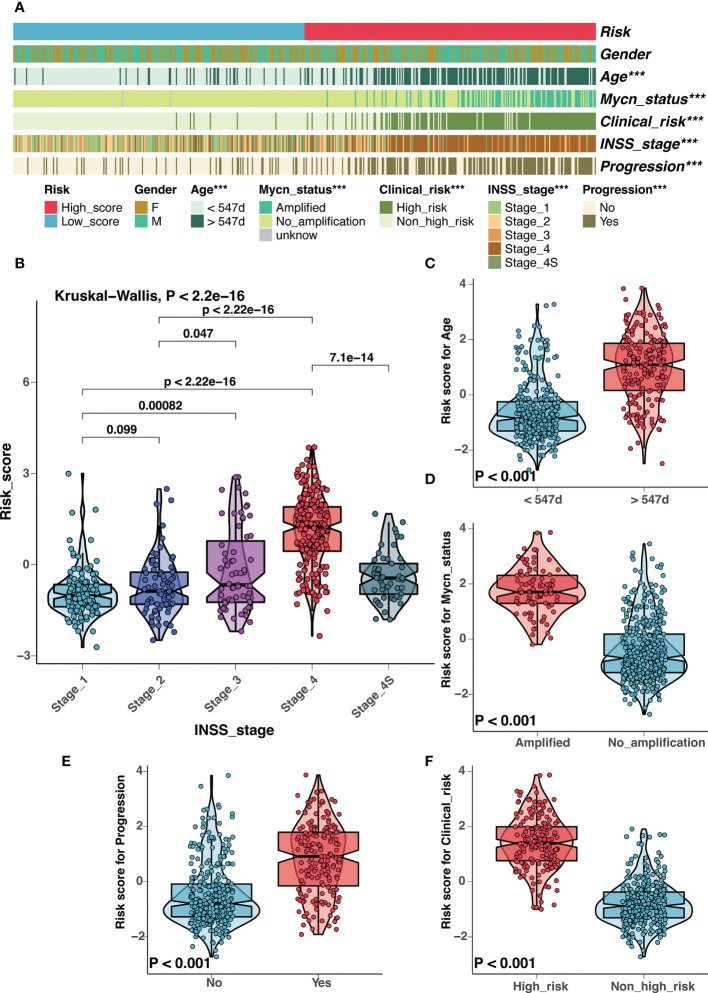
Correlation analysis between clinical characteristics and the risk signature in GSE49710 cohort. **(A)** Correlation analysis between the risk signature and clinical characteristics in GSE49710 cohort. **(B-F)** The comparison of risk scores between samples with different clinical characteristics, including INSS stage **(B)**, age **(C)**, MYCN status **(D)**, progression **(E)**, and COG risk groups **(F)**. (***: P < 0.001).

There had been several established prognostic factors in neuroblastoma, including age, INSS stage, MYCN status, and the clinical risk classification system. We explored the correlation between the risk signature and these prognostic factors. As illustrated in **Figure 6A**, the risk score was significantly associated with age, MYCN amplification, clinical risk group, stage, and progression. Patients with unfavorable clinical characteristics present high scores (**Figures 6B-F**). Similar results were observed in the Tianjin cohort, and there were significant high scores in samples with advanced stage and unfavorable histology (**Figure S7**).

**Figure 7:**
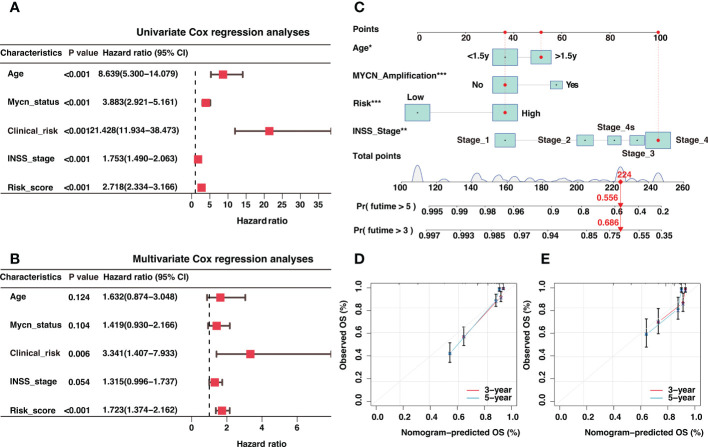
Independent prognosis analysis of the risk signature and construction of a nomogram for 3-year and 5-year overall survival (OS) prediction. **(A)** The univariate Cox regression analysis in GSE49710 cohort **(B)**. The multivariate Cox regression analysis in GSE49710 cohort. **(C)** The establishment of a nomogram that predicted 3-year and 5-year OS in the GSE49710 cohort. **(D, E)** Calibration curves of the nomogram in the prediction of 3-year and 5-year OS in GSE49710 **(D)** and E-MTAB-8248 **(E)** cohorts. *: P < 0.05, **: P < 0.01, ***: P < 0.001.

In light of the correlation between the risk signature and clinical characteristics, we investigated the independent prognostic significance of the risk signature. After univariate and multivariate cox regression analysis, the risk signature was identified as an independent prognostic factor in the GSE4910 (**Figures 7A, B**), E-MTAB-8248 (**Figure S8A**), and TARGET (**Figure S8B**) cohorts, respectively. To optimize the clinical utilization in individual prognosis prediction, we incorporated the risk signature and several clinical risk factors to construct a nomogram in the GSE49710 cohort (**Figure 7C**). The nomogram could assign a score to each prognostic factor and predict 3-year and 5-year OS based on the sum of scores in each sample (**Figure 7C**). The calibration curves were plotted to evaluate the accuracy of the nomogram, and the nomogram prediction curves were quite close to standard curves in GSE49710 (**Figure 7D**), E-MTAB-8248 (**Figure 7E**), and TARGET (**Figure S8C**) cohorts, suggesting an excellent accuracy for prognosis prediction in all datasets. The nomogram incorporated the risk score and multiple established prognostic factors and could precisely predict neuroblastoma prognosis.

### 3.4 Immune landscape of the ganglioside-related risk signature

The gene set enrichment analysis (GSEA) was conducted to elucidate the biological functions behind the variations in prognosis between low-score and high-score groups. As shown in **Figure S9A**, the high-score group was significantly enriched in tumorigenic pathways, including cell cycle, DNA replication, homologous recombination, ribosome, and spliceosome. Interestingly, the low-score group was enriched in antigen processing and presentation and cell adhesion pathways, implying underlying immune landscape differences between the two groups (**Figure S9B**).

Impairment of the antigen-presenting machinery (APM) contributed heavily to the low immunogenicity of neuroblastoma. It was widely accepted that antigen presentation through MHC-I molecules did not function in neuroblastoma due to low expression levels (20, 39). These characteristics made neuroblastoma cells almost undetectable to CD8 T cells. Interestingly, the low-score group was enriched in the antigen processing and presentation pathway, implying a potential role of ganglioside in APM of neuroblastoma.

**Figure 8:**
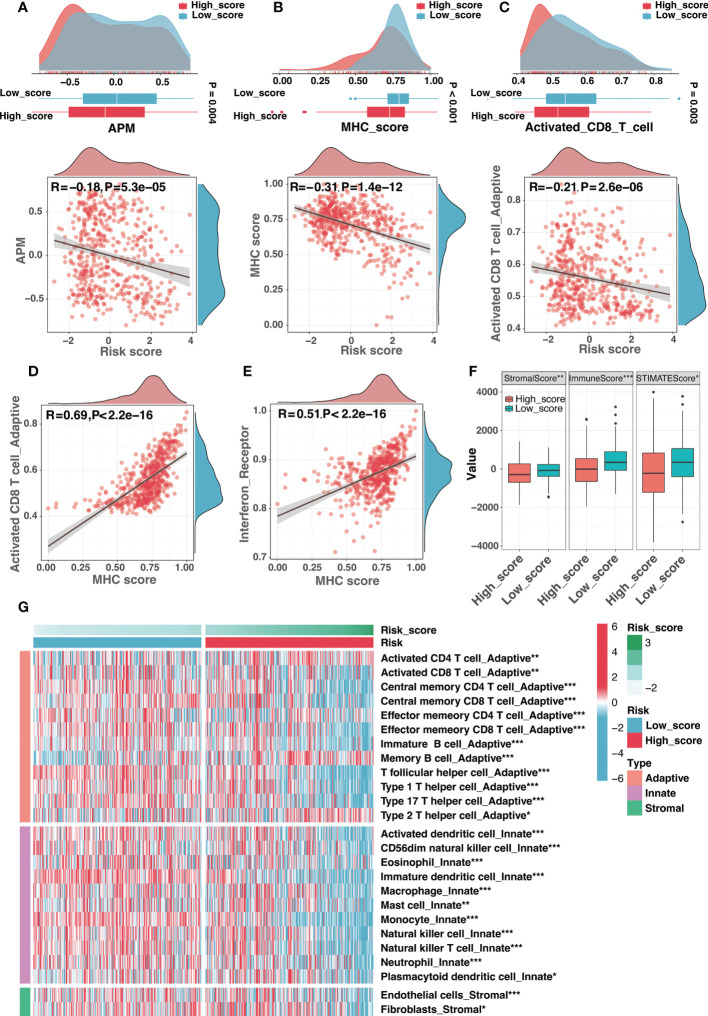
The immune landscape of the risk signature. **(A)** The comparison of antigen-presenting machinery (APM) score between different risk groups (top) and correlation analysis between the risk score and the APM score (bottom). **(B)** The comparison of MHC score between different risk groups (top) and correlation analysis between the risk score and the MHC score (bottom). **(C)** The comparison of activated CD8 T cell infiltration between different risk groups (top) and correlation analysis between the risk score and the activated CD8 T cell infiltration (bottom). **(D, E)** The correlation analysis between the MHC score and the activated CD8 T cell infiltration **(D)** as well as interferon receptor activity **(E)**. **(F)** Comparison of the stromal, immune, and ESTIMATE scores between high-score and low-score groups. **(G)** Different infiltration levels of immune infiltrating cells between low-score and high-score groups. (*:P< 0.05, **: P < 0.01, ***: P < 0.001).

We introduced the APM scores (APS) developed in previous research (detailed in “Materials and Methods”) as a proxy for antigen processing and presentation efficiency. As seen in **Figure 8A**, the APS was significantly and adversely linked with the risk score, showing that APM in the high-score group was suppressed (**Figure 8A**). Considering MHC-I molecules as a critical component of APM, the MHC score (described in “Materials and Methods”) was developed using ssGSEA to assess MHC-I activity. As expected, there was a strong negative association between risk score and MHC score as well as activated CD8 T cell infiltration (**Figures 8B, C**), and increased MHC-I activity was associated with high activated CD8 T cell infiltration (**Figure 8D**). Additionally, consistent with the concept that IFNγ could increase MHC-I expression in neuroblastoma (40), the MHC score was substantially and positively correlated with the activity of interferon receptors (**Figure 8E**). In general, APM impairment contributed significantly to immune escape in the high-score group, and the low activity of MHC-I may be an important cause.

Moreover, the absence of leukocytes and the presence of immunosuppressive myeloid and stromal cells were also efficient strategies for tumor immune evasion in neuroblastoma. Interestingly, ganglioside soluble GD2 could reduce T cell proliferation, suggesting the role of ganglioside in tumor immune evasion of neuroblastoma. The ESTIMATE method was used to determine the infiltration levels of immune cells and stromal cells. The high-score group presented significantly lower stromal and immune scores than the low-score group (**Figure 8F**). As illustrated in **Figure 8G**, most immune infiltrating cells were infiltrated at low levels in the high-score group, demonstrating an immune escape mechanism resulting from the lack of immune cells in the high-score group. In summary, the immune escape strategy in the high-score group was partially attributed to the lack of immune cell infiltration.

**Figure 9:**
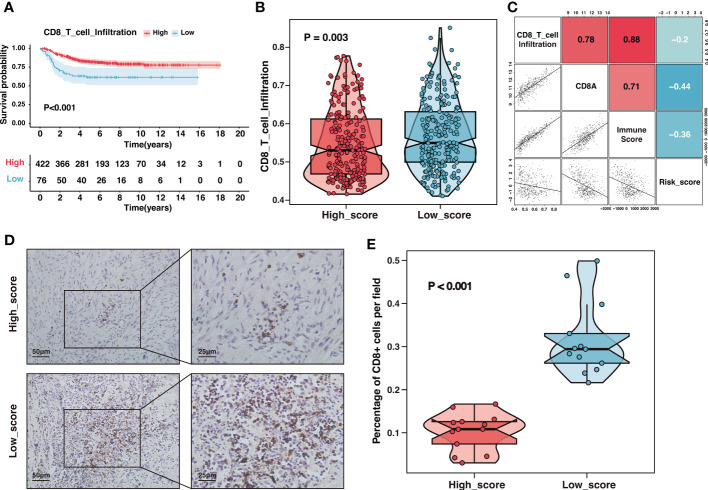
The high-score group presented low CD8^+^ T-cell infiltration. **(A)** Kaplan–Meier curves of overall survival between samples with different infiltration of CD8^+^ T-cell in the GSE49710 dataset. **(B)** The comparison of CD8^+^ T-cell infiltration between high-score and low-score groups in the GSE49710 dataset. **(C)** The correlation analysis of the risk score, CD8^+^ T-cell infiltration, CD8A expression and immune-score in the GSE49710 dataset. **(D)** Representative immunohistochemistry (IHC) images showing the infiltration of CD8^+^ T-cell between different risk groups in Tianjin cohort. Magnification, ×200, ×400. **(E)** The comparison of CD8^+^ T-cell infiltration between different risk groups in Tianjin cohort.

Tumor-infiltrating lymphocytes were essential constituents of the tumor immune microenvironment in neuroblastoma. Recent studies revealed reduced CD8^+^ T lymphocyte infiltration in high-risk and advanced-stage neuroblastoma (20). Children with a rising rate of CD8^+^ T lymphocytes had a better prognosis, highlighting that strengthening CD8^+^ T-cell responses would be a promising therapy opportunity (41). Consistent with previous studies, our results showed that samples with high CD8^+^ T lymphocyte infiltration presented an excellent prognosis in the GSE49710 cohort (**Figure 9A**). Furthermore, the risk signature was a potent indicator of CD8^+^ T lymphocyte infiltration. Compared to samples with a high score, samples with a low score displayed a significantly greater infiltration of CD8^+^ T-cells (**Figure 9B**). The risk score was adversely and significantly associated with CD8^+^ T-cell infiltration and CD8A expression (**Figure 9C**). We further performed immunohistochemistry on tissue samples from the Tianjin cohort to confirm this finding. As indicated in **Figures 9D, E**, there was a considerable decrease in CD8^+^ T-cell infiltration in the high-score group compared to the low-score group (**Figures 9D, E**). In summary, the risk signature could reliably predict CD8^+^ T-cell infiltration in the immune microenvironment of neuroblastoma.

**Figure 10:**
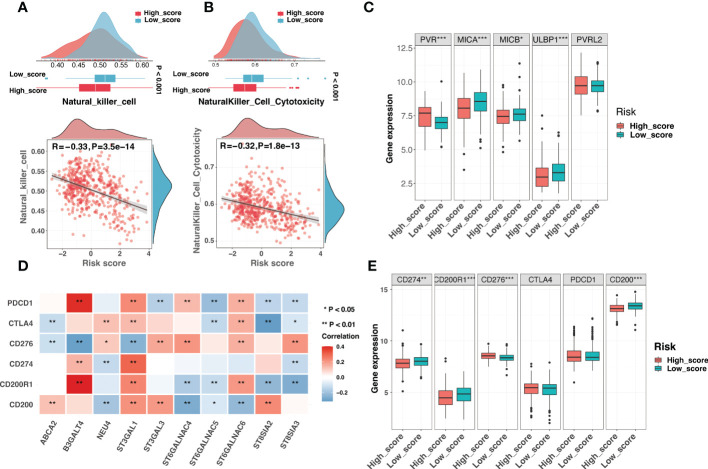
Comparison of NK cell activity and immune checkpoints in the risk signature **(A)** The comparison of the NK cell infiltration level between different risk groups (top) and correlation analysis between the risk score and the NK cell infiltration level (bottom). **(B)** The comparison of the NK cell cytotoxicity between different risk groups (top) and correlation analysis between the risk score and the NK cell cytotoxicity (bottom). **(C)** The boxplot exhibited different expression levels of ligands for NK cell-activating receptors between low-score and high-score groups. **(D)** The correlation analysis between the expression of signature genes and established immune checkpoints. **(E)** The boxplot demonstrated different expression levels of immune checkpoints between low-score and high-score groups. (*: P < 0.05, **: P < 0.01, ***: P < 0.001).

It had been shown that gangliosides were involved in regulating NK cell cytotoxicity through multiple mechanisms (42, 43). Importantly, antibody-dependent cell-mediated cytotoxicity (ADCC) mediated by NK cells and neutrophils was a critical mechanism for anti-GD2 antibody efficacy (44). Neuroblastoma cells lacking MHC-I molecules, which served as the ligands for killer inhibitory receptors, should be particularly susceptible to NK cells. Notably, the high-score group with reduced MHC-I activity exhibited limited NK cell infiltration and cytotoxicity (**Figures 10A, B**). NK-mediated elimination of neuroblastoma seemed to be shielded by other mechanisms that modify the balance of activating and inhibitory signals on NK cells (20). Therefore, we compared the expression of ligands for NK-activating receptors between two risk groups. Apart from PVR, ligands for the NK cell-activating receptors DNAM-1 and NKG2D, such as MICA, MICB, and ULBP1, were downregulated in the high-score group (**Figure 10C**). The downregulation of NK-activating receptors may be the potential reason for the inhibition of NK cells. Taken together, escaping NK cells was an essential driver of immunosuppression in the high-score group.

Immune checkpoints (ICs) in the tumor microenvironment could significantly impact the reactivity of tumor-infiltrating lymphocytes to neuroblastoma (20). Signature genes were significantly associated with several ICs, indicating that ICs may play a role in the ganglioside-related risk signature (**Figure 10D**). As seen in **Figure 10E**, the low-score group had increased expression of PD-L1 (CD274), CD200R1, and CD200, while the high-score group had increased expression of B7-H3 (CD276). Briefly, immune evasion strategies were mediated by different ICs in low-score and high-score groups.

### 3.5 The ganglioside-related risk signature could predict immunotherapeutic response and chemotherapy sensitivity

**Figure 11:**
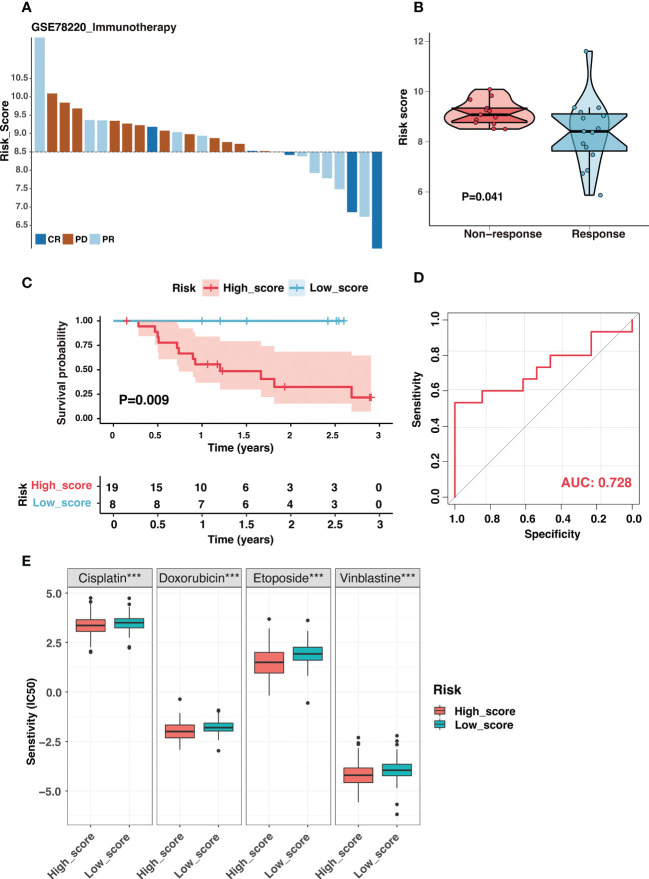
The ganglioside-related risk signature could predict immunotherapeutic response and chemotherapy sensitivity. **(A)** The distribution of immunotherapy responses at different risk scores in the GSE78220 cohort. **(B)** The comparison of risk scores between non-response and response groups in the GSE78220 cohort. **(C)** Kaplan–Meier curves of overall survival in GSE78220 cohort between different risk groups. **(D)** The receiver operating characteristic (ROC) curves for immunotherapy response prediction in the GSE78220 cohort. **(E)** The boxplot demonstrated different IC50 (the half maximal inhibitory concentration) values of four chemotherapeutic drugs, including cisplatin, doxorubicin, etoposide and vinblastine, between low-score and high-score groups. (***: P < 0.001).

Immune checkpoint inhibitors (ICIs) had revolutionized cancer treatment. However, only a small number of patients were responding (45). Considering the dramatic differences in the immune landscape between the two groups, we investigated the risk signature’s predictive ability for immunotherapeutic benefits. Due to the lack of expression data for immunotherapy of neuroblastoma, we used the immunotherapy dataset of melanoma, which is also a neuroendocrine tumor, to investigate the role of the risk signature in immunotherapeutic response prediction. **Figure 11A** depicted the distribution of treatment response at different risk scores in GSE78220, and the risk score was significantly lower in the immunotherapy-responsive group than in the non-responsive group (**Figure 11B**). Additionally, samples with high scores had a poor prognosis (**Figure 11C**). The risk signature had excellent discrimination in predicting immunotherapy response, presenting an AUC of 0.728 (**Figure 11D**). In short, our findings showed that the ganglioside-related risk signature could accurately predict responsiveness to immune checkpoint blockade therapy. The low-score group showed a better response to immunotherapy than the high-score group.

Chemotherapy was the cornerstone of neuroblastoma treatment, and we examined the susceptibility of the two groups to commonly used chemotherapeutic drugs in the established treatment regimen. The IC50 values of four chemotherapeutic drugs were compared between two groups: cisplatin, doxorubicin, etoposide, vinblastine. Interestingly, the IC50 values for these chemotherapeutic drugs were significantly lower in the high-score group (**Figure 11E**). Function enrichment in the cell cycle and DNA replication of the high-score group may be the potential reason (**Figure S9**). These results suggested that the high-score group could still benefit from the established chemotherapy regime.

### 3.6 The high-score group presented with a high degree of dedifferentiation

**Figure 12:**
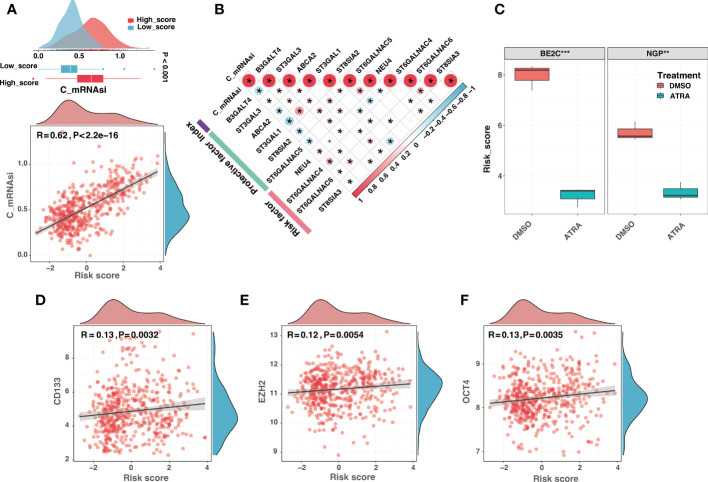
The high-score group presented with a high degree of dedifferentiation. **(A)** The comparison of the corrected mRNAsi (c_mRNAsi) between different risk groups (top) and correlation analysis between the risk score and the c_mRNAsi (bottom). **(B)** The correlation analysis between the c_mRNAsi and signature gene expression. **(C)** The comparison of risk scores between neuroblastoma cells treated with dimethyl sulfoxide (DMSO) or all-trans retinoic acid (ATRA) in BE2C and NGP cell lines. **(D-F)** The risk score was significantly positively associated with multiple stemness markers of neuroblastoma, including CD133 **(D)**, EZH2 **(E)**, and OCT4 **(F)**. (*: P < 0.05, **: P < 0.01, ***: P < 0.001).

The previous study showed that ganglioside was implicated in maintaining neural stem cell self-renewal capacity (15). Furthermore, the critical implications of gangliosides in tumor stem cells have been frequently highlighted (16, 46–48). Recent research suggested that the low MHC-I expression of neuroblastoma may reflect the undifferentiated state of the neural crest (20). These findings implied that gangliosides might contribute to the undifferentiated state of neuroblastoma. Interestingly, DEGs between ganglioside-related clusters were highly enriched in neural crest cell development (**Figure S4**). Therefore, we explored the role of ganglioside-related risk signature in reflecting the degree of neuroblastoma dedifferentiation. Firstly, we investigated the connection between the risk score and the corrected stemness index mRNAsi (c_mRNAsi). **Figure 12A** showed a significant positive correlation between the risk score and c_mRANsi (R = 0.62, P < 0.001). Similarly, all signature genes were significantly correlated with the c_mRNAsi (**Figure 12B**). We subsequently validated this result in a dataset of neuroblastoma cell lines. It had been known that all-trans retinoic acid (ATRA) could induce differentiation in both primary neuroblastomas and cell lines. As shown in **Figure 12C**, neuroblastoma cells treated with ATRA exhibited a reduced risk score in both BE2C and NGP cell lines. In addition, the risk score was significantly positively associated with multiple stemness markers of neuroblastoma, including CD133, EZH2, and OCT4 (**Figures 12D-F**). These results indicated that the high-score group presented with a high degree of dedifferentiation.

### 3.7 Downregulated B3GALT4 promoted the progress of neuroblastoma cells

**Figure 13:**
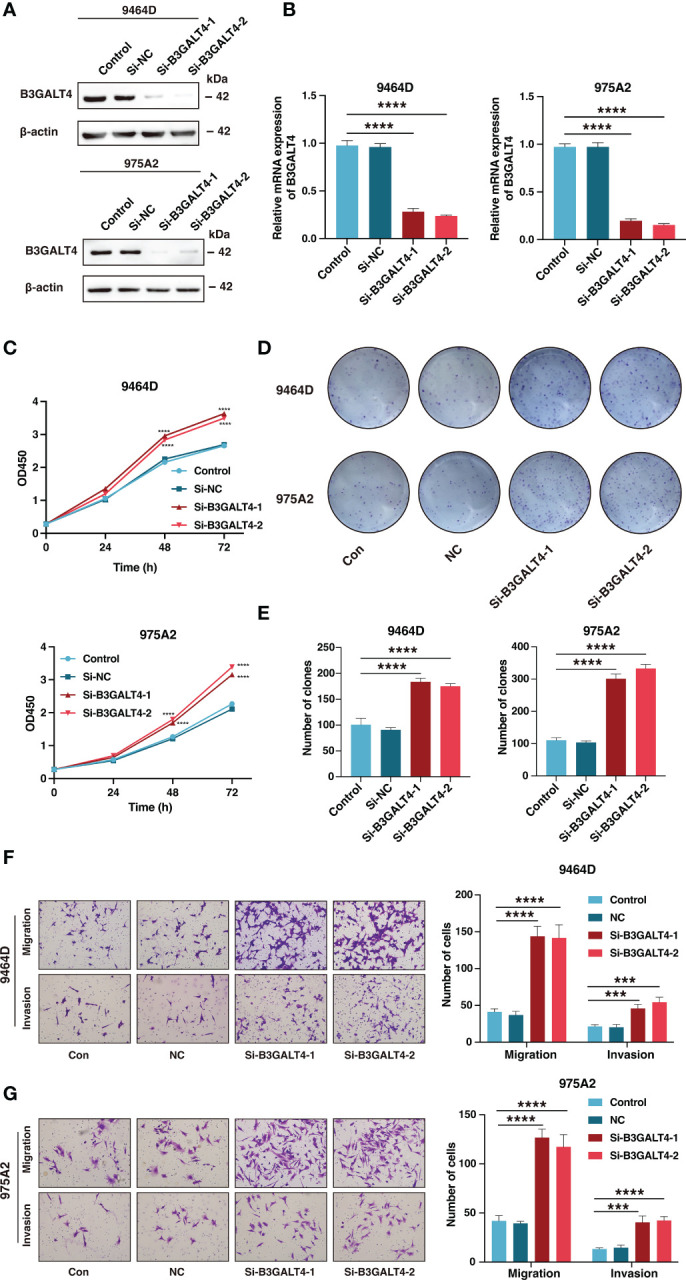
The downregulation of B3GALT4 could promote the progression of neuroblastoma. **(A, B)** The western blot analysis **(A)** and quantitative real-time PCR **(B)** was performed to validate the downregulation of B3GALT4 after transfection with siRNA in 9464D and 975A2 cells. **(C)** The CCK-8 assay was performed to measure the proliferation capacity of 9464D and 975A2 cells. **(D, E)** The colony formation assay **(D)** and corresponding statistical analysis **(E)** of 9464D and 975A2 cells. **(F, G)** The transwell assays were conducted to determine the effect of down-regulated B3GALT4 on neuroblastoma migration and invasion capacity in 9464D **(F)** and 975A2 cells **(G)**. (***, P< 0.001; ****, P< 0.0001).

Considering B3GALT4 as the connecting link of ganglioside and with the highest absolute value of regression coefficient in signature genes, the siRNA of B3GALT4 was transfected into 9464D and 975A2 cells, and the role of B3GALT4 in neuroblastoma was explored. The western blot and RT-qPCR were performed to verify the downregulation of B3GALT4 expression after transfection for further experiments (**Figures 13A, B**). As shown in **Figure 13C**, the CCK-8 assay demonstrated the knockdown of B3GALT4 significantly promoted cell proliferation in both cell lines (**Figure 13C**). Consistently, colony formation assay showed that neuroblastoma cells with downregulation of B3GALT4 present more cell clones than the control group in both cell lines (**Figures 13D, E**). Besides, cells detected in the lower chamber were significantly increased after transfected with si-B3GALT4 compared to the control group in both invasion and migration assays (**Figures 13F, G**). Our findings suggested that B3GALT4 could inhibit the progression of neuroblastoma, verifying the protective role of B3GALT4 in the risk signature.

## 4 Discussion

Neuroblastoma is an extraordinarily lethal childhood tumor characterized by high heterogeneity. Precise and efficient prognostic prediction is critical to guide treatment. In the present study, we identified two ganglioside-related clusters with differential expression patterns and outcomes, indicating a substantial prognostic significance of gangliosides in neuroblastoma. To effectively predict the individualized prognosis of neuroblastoma based on gangliosides, we conducted the Lasso-penalized Cox regression analysis on ganglioside-related DEGs to identify independent prognostic genes and developed a ten-gene risk signature. The risk signature showed excellent discrimination and accuracy in GSE49710, TARGET, E-MTAB-8248, and Tianjin cohorts. Additionally, the risk signature was significantly related to several previously identified prognostic markers and was demonstrated to be an independent prognostic factor for neuroblastoma. Besides, a nomogram incorporating multiple established clinical prognostic characteristics was developed and verified. In summary, we developed a novel ganglioside-related risk signature in neuroblastoma that enables reliable and individualized prognosis prediction.

There are ten genes in the ganglioside-related risk signature. However, few of them are identified as prognostic genes in neuroblastoma. ABCA2 encodes a membrane-associated protein belonging to the ATP-binding cassette transporter superfamily and is involved in the metabolism of gangliosides (49). Additionally, ABCA2 is overexpressed in pediatric acute lymphoblastic leukemia and may contribute to multidrug resistance (50). B3GALT4 is involved in synthesizing GM1/GD1 gangliosides and has been identified as a prognostic marker for osteosarcoma and neuroblastoma (51, 52). Our results also showed the inhibition of B3GALT4 could significantly increase neuroblastoma cell proliferation, migration, and invasion *in vitro*. Notably, NEU4 has been identified as a potential regulator of neuronal development, with overexpression promoting the acquisition of a stem cell-like phenotype in neuroblastoma cells (53). The sialyltransferases are required to synthesize gangliosides, and their aberrant expression is closely related to a poor prognosis in tumors (54, 55). Among them, ST3GAL1 overexpression promotes epithelial-mesenchymal transition, migration, and invasion in ovarian cancer (56), and ST3GAL3 downregulation inhibits pancreatic cancer cell migration and invasion (57). Additionally, the sialyltransferases ST6GalNAc4, ST6GalNAc5, and ST6GalNAc6 contribute to the synthesis and metabolism of the gangliosides GD1a and GM1b. Overexpression of ST6GalNAc4 has been crucial for tumor cell glycosylation modification and lung cancer metastasis, although the roles of ST6GalNAc5 and ST6GalNAc6 in malignancies remain unclear (55). Furthermore, the polysialyltransferase ST8SIA2 is also implicated in small cell lung cancer and glioma metastasis and invasion (55). Consistent with ST8SIA3 as a risk factor in the present study, it mediates the sialylation of GM3 and GD3 and promotes survival, proliferation, clonogenicity, and migration of glioblastoma cells (55). To summarize, these signature genes are intimately engaged in the synthesis and metabolism of gangliosides and play a critical role in tumor development and progress, supporting the predictive value of the risk signature in neuroblastoma.

Our result revealed that the low-score group was enriched in the antigen processing and presentation pathway, implying potential differences in the immune microenvironment between the two groups. Gangliosides have been identified as potent inhibitors of the cellular immune response. Soluble GD2 shed from neuroblastoma cells has been shown to suppress T cell proliferation and contribute to tumor immune evasion in neuroblastoma (8, 13, 20). Moreover, anti-GD2 antibodies have been introduced into the standard treatment regime for high-risk neuroblastoma. Given the critical role of gangliosides in the neuroblastoma immune microenvironment, we investigated and compared the immune landscape between the low-score and high-score groups.

Neuroblastoma has low immunogenicity, characterized by a low mutational burden and abnormalities in the antigen processing and presenting machinery. Our findings indicated that the high-score group exhibited an impairment in APM and low MHC-I activity, suggesting that the high-score group was less immunogenic than the low-score group. Interestingly, increasing researches indicate that the fundamental reason for MHC-I suppression may be the embryonic origin of neuroblastoma. Neuroblastoma seems to represent the underdeveloped neural crest state, characterized by low MHC-I expression. Neuroblastoma cell line differentiation is associated with increased MHC-I expression (20, 58). In the present study, low MHC-I expression in the high-score group may represent a substantial degree of dedifferentiation. High c_mRNAsi in the high-score group supports this hypothesis. We also found that ATRA-induced differentiated neuroblastoma cell lines exhibited reduced scores. The risk score was also significantly positively associated with multiple stemness markers of neuroblastoma. Generally, these results suggested that gangliosides may play a role in the formation and dedifferentiation of neuroblastoma, and the ganglioside-related risk signature could reflect the degree of neuroblastoma dedifferentiation.

Low immunogenicity leads to insufficient infiltration of lymphocytes into the tumor and poor anti-tumor reactivity (20). The high-score group with low immunogenicity showed significantly reduced immune scores and infiltration levels. The CD8^+^ T-cell, a crucial component of the immune response to tumors, has been identified as one of the most significant immunotherapy targets for tumors (59). Recent research has demonstrated that CD8^+^ T-cell infiltration is substantially related to neuroblastoma prognosis (20). We discovered that the risk signature could predict CD8^+^ T-cell infiltration accurately and validated it using tissue samples in the Tianjin cohort. Interestingly, several immunosuppressive and stromal cells were substantially infiltrated in the low-score group. ST3GAL1 has been implicated in transforming tumor-associated macrophage differentiation to a more suppressive phenotype (55). Consistently, our results also showed that the expression level of ST3GAL1 was significantly upregulated in the low-score group. Immunosuppressive cell infiltration represents a potential immune escape mechanism in the low-score group, and targeting these cells may be an effective immunotherapeutic strategy.

Generally, the cytotoxic activity of NK cells is inhibited by the binding of killer-cell immunoglobulin-like receptors to MHC-I molecules on normal cells. In contrast, tumor cells typically lack MHC-I and are thus vulnerable to NK-mediated killing (20, 39, 60). Surprisingly, the high-score group with low MHC-I activity had a low NK cell infiltration and cytotoxicity level, indicating a potent NK cell suppression in this group. The imbalance of NK cell activation and inhibitory signaling is a critical mechanism of immune escape in neuroblastoma (20, 39). We found ligands for NK cell activating receptors, including MICA, MICB, and ULBP1 were downregulated in the high-score group. The inhibition of NK cell activation may be the potential reason for escaping NK cells in the high-score group. Additionally, previous research showed that overexpression of B7-H3 molecules could inhibit NK cell cytotoxicity, and we found that the high-score group exhibited increased B7-H3 expression (60). The escaping NK cell significantly contributes to immunosuppression in the high-score group. Fortunately, the anti-GD2 antibody Dinutuximab could restore the NK cell balance and promote NK cell cytotoxicity against neuroblastoma potently, highlighting the need for anti-GD2 immunotherapy in the high-score group (20).

The presence of immune checkpoints is a critical mechanism by which cancers escape the immune system, and immune checkpoint inhibitors have made breakthroughs in adult malignancies. However, the efficacy of ICIs in neuroblastoma is not satisfactory, and identifying individuals who respond to immunotherapy is essential. We found that PD-L1 was significantly overexpressed in the low-score group, indicating that the low-score group may benefit from ICIs (20). The ganglioside-related risk signature could effectively predict immunotherapy response in the ICI-treated cohort, with better response to immunotherapy in the low-score group. Therefore, ICIs are potential options for immunotherapy in the low-score group, further verification by large-scale and multi-center investigations are required in neuroblastoma.

There are some limitations to this study. There is a shortage of molecular sequencing data for anti-GD2 therapy in neuroblastoma, and the risk signature for predicting anti-GD2 antibody immunotherapy response requires additional confirmation. It is encouraging to note that the anti-GD2 immunotherapy for neuroblastoma has been introduced in China, and a relevant clinical trial is in progress in our institution (CTR20221154). Furthermore, since this study is based on retrospective data, it requires further validation from a comprehensive perspective research. The treatment strategies that we advocated for the different subgroups in the risk signature, including chemotherapy and immunotherapy, should be assessed in prospective clinical trials. Additionally, thorough laboratory investigations are necessary to elucidate the comprehensive biological functions of signature genes.

## 5 Conclusion

In this work, we developed a novel ganglioside-related risk signature that enabled precise prognostic prediction of neuroblastoma. Additionally, the risk signature identified distinct immune landscapes and immune evasion strategies between risk groups and could be used to predict immunotherapy response. This work emphasizes the critical role of gangliosides in the prognosis and immune microenvironment of neuroblastoma, which may inform clinical evaluation and therapeutic decision-making.

## Data availability statement

Publicly available datasets were analyzed in this study. The names of the repositories and accession numbers can be found within the article/**Supplementary Materials**.

## Ethics statement

The studies involving human participants were reviewed and approved by the Ethics Committee of Tianjin Medical University Cancer Institute and Hospital (E20210027). Written informed consent to participate in this study was provided by the participants’ legal guardian/next of kin.

## Author contributions

LH and QZ designed the study. JY, LH, YS, YJ, ZL, BG, JL, YL, and YW analyzed and interpreted data. JY drafted the manuscript, and major revised by LH and QZ. All authors contributed to the article and approved the submitted version.

## Supplementary material

Figure S1 The workflow of the present study.

Figure S2 Consensus clustering analysis based on differentially expressed ganglioside-related genes in the GSE49710 dataset. (A-H) Consensus score matrix of samples when k = 2-9. (I) CDF of the consensus matrix for each k (indicated by colors). (J) Relative alterations in the area under CDF curves. (K) Tracking plot for each k.

Figure S3 Consensus clustering analysis based on differentially expressed ganglioside-related genes in the TARGET dataset. (A-H) Consensus score matrix of samples when k = 2-9. (I) CDF of the consensus matrix for each k (indicated by colors). (J) Relative alterations in the area under CDF curves. (K) Tracking plot for each k. (L) The t-SNE analysis revealed a clear distinction between the two clusters in the TARGET cohort. (M) Kaplan–Meier curves of overall survival in TARGET cohort between different ganglioside-related clusters.

Figure S4 Gene Ontology (GO) and Kyoto Encyclopedia of Genes and Genomes (KEGG) functional enrichment analysis of differentially expressed genes between ganglioside-related clusters A and B (indicated by colors).

Figure S5 Validation of the ganglioside-related risk signature. (A) The distribution of the risk score (top) and survival time (bottom) in the E-MTAB-8248 cohort. (B) The receiver operating characteristic (ROC) curves of the risk signature for 3-year and 5-year OS prediction in the E-MTAB-8248 cohort. (C) The ROC curves of clinical prognostic factors for 3-year OS prediction in the E-MTAB-8248 cohort. (D) Kaplan–Meier curves of overall survival (OS) in the E-MTAB-8248 cohort between different risk groups. (E) The distribution of the risk score (top) and survival time (bottom) in the TARGET cohort. (F) The ROC curves of the risk signature for 3-year and 5-year OS prediction in the TARGET cohort. (G) The ROC curves of clinical prognostic factors for 3-year OS prediction in the TARGET cohort. (H) Kaplan–Meier curves of overall survival (OS) in the TARGET cohort between different risk groups. (I-K) The principal component analysis (PCA) of the risk signature in GSE49710 (I), E-MTAB-8248 (J), and TARGET (K) cohorts.

Figure S6 The prognostic value of the ganglioside-related risk signature in samples in COG high-risk group or older than 18 months. (A-B) Kaplan–Meier curves of samples in COG high-risk group in GSE49710 (A) and TARGET (B) cohorts. (C-D) Kaplan–Meier curves of samples older than 18 months in GSE49710 (C) and TARGET (D) cohorts.

Figure S7 Correlation analysis between clinical characteristics and the risk signature in the Tianjin cohort. (A-E) The comparison of risk scores between samples with different clinical characteristics in the Tianjin cohort, including INRGSS stage (A), MYCN status (B), COG risk groups (C), pathology (D), and INSS stage (E).

Figure S8 Independent prognosis analysis of the risk signature and external validation of the nomogram. (A-B) The univariate and multivariate Cox regression analysis in E-MTAB-8248 (A) and TARGET (B) cohorts. (C) Calibration curves of the nomogram in the prediction of 3-year and 5-year overall survival (OS) in the TARGET cohort.

Figure S9 Gene set enrichment analysis (GSEA) of the risk signature. The GSEA in the high-score (A) and low-score (B) groups.
