# Supplementary material for: A novel ganglioside-related risk signature can reveal the distinct immune landscape of neuroblastoma and predict the immunotherapeutic response
Source: Front Immunol. 2022 Dec 20;13:1061814. doi: 10.3389/fimmu.2022.1061814 (PMC9807785; doi:10.3389/fimmu.2022.1061814)
Supplement: Figure S1 — The workflow of the present study. [file DataSheet_1.zip › Supplementary material/TableS11.docx]

Table S11 Clinical baseline characteristics of samples in Tianjin cohort

| Characteristics | N (%) |
| --- | --- |
| Gender |  |
| Male | 12 (46.2) |
| Female | 14 (53.8) |
| Age |  |
| <18 months | 2 (7.7) |
| ≥18 months | 24 (92.3) |
| INSS Stage |  |
| 1 | 5 (19.2) |
| 2 | 4 (15.4) |
| 3 | 2 (7.7) |
| 4 | 15 (57.7) |
| INRGSS Stage |  |
| L1 | 9 (34.6) |
| L2 | 2 (7.7) |
| M | 15 (57.7) |
| INPC |  |
| FH | 10 (38.5) |
| UH | 16 (61.5) |
| MYCN Status |  |
| Not amp | 16 (61.5) |
| Amp | 7 (26.9) |
| Unknow | 3 (11.5) |
| COG risk group |  |
| Low-risk | 8 (30.8) |
| High-risk | 18 (69.2) |

INSS, International Neuroblastoma Staging System; INRGSS, International Neuroblastoma Risk Group Staging System; INPC, International Neuroblastoma Pathology Classification; FH, favorable histology; UH, unfavorable histology; Amp, amplified; COG, Children's Oncology Group
