# Supplementary figures and images for: A novel ganglioside-related risk signature can reveal the distinct immune landscape of neuroblastoma and predict the immunotherapeutic response
Source: Front Immunol. 2022 Dec 20;13:1061814. doi: 10.3389/fimmu.2022.1061814 (PMC9807785; doi:10.3389/fimmu.2022.1061814)

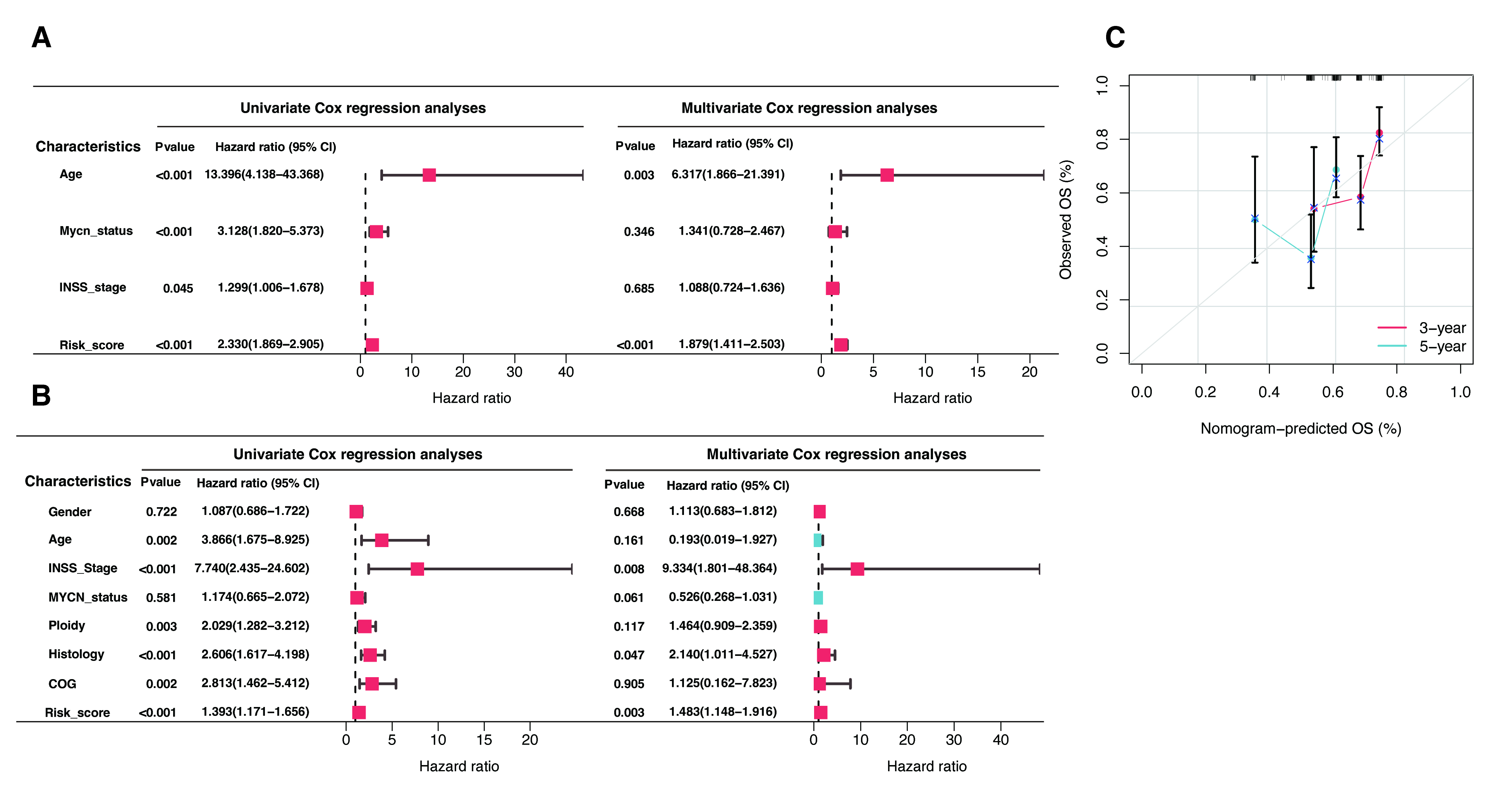

Supplement: Figure S1 — The workflow of the present study. [file DataSheet_1.zip › Supplementary material/FigureS8.tif]

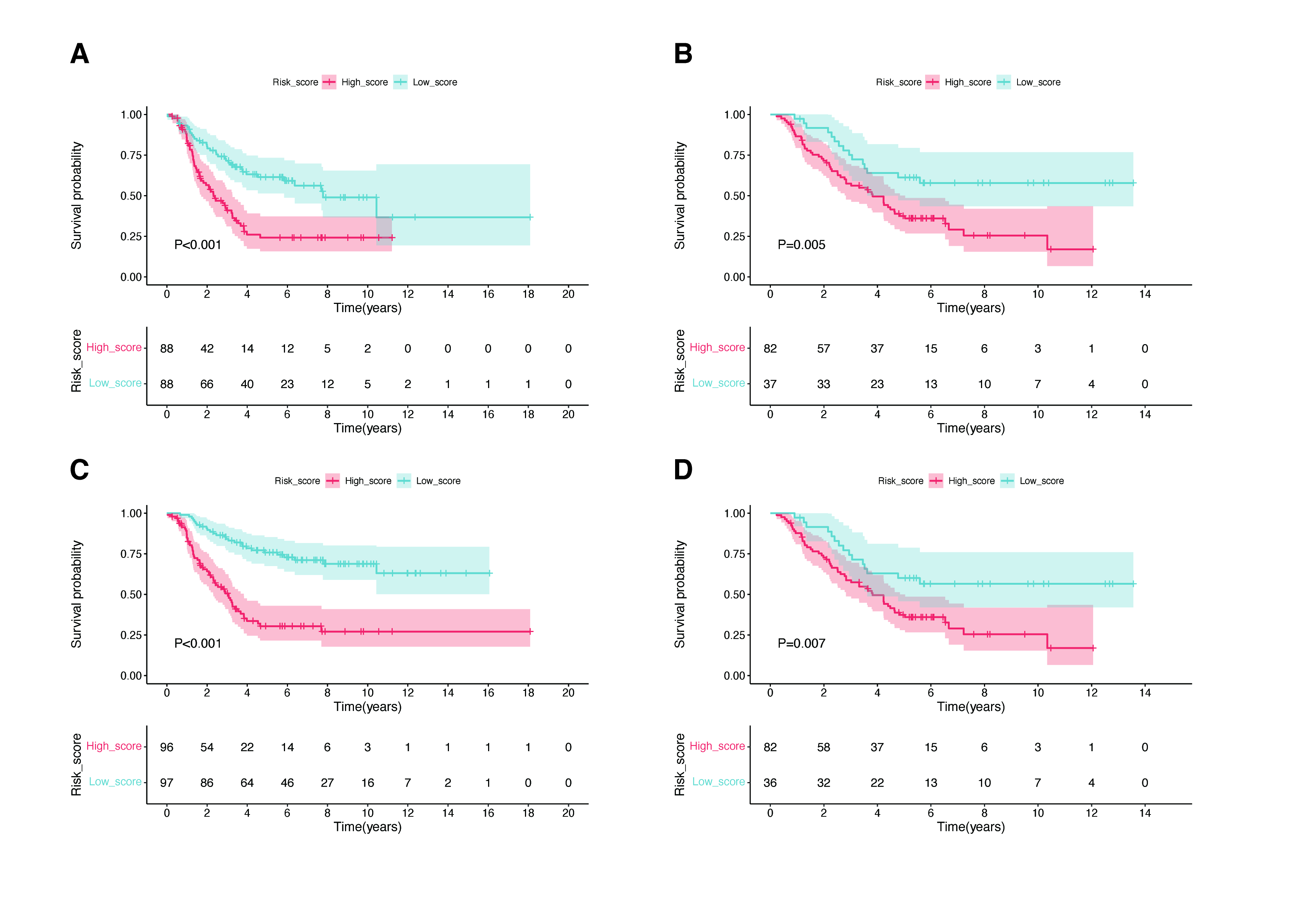

Supplement: Figure S1 — The workflow of the present study. [file DataSheet_1.zip › Supplementary material/FigureS6.tif]

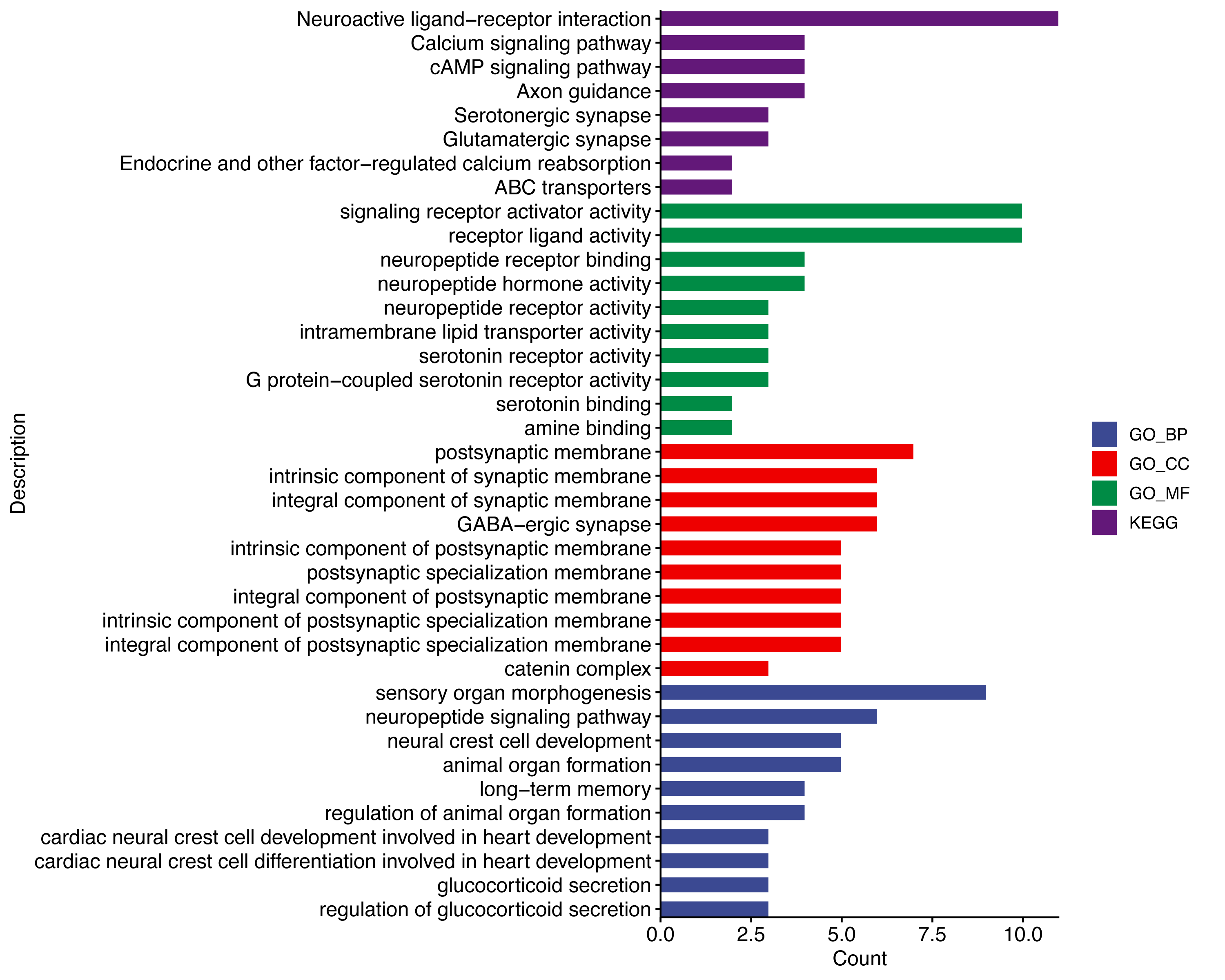

Supplement: Figure S1 — The workflow of the present study. [file DataSheet_1.zip › Supplementary material/FigureS4.tif]

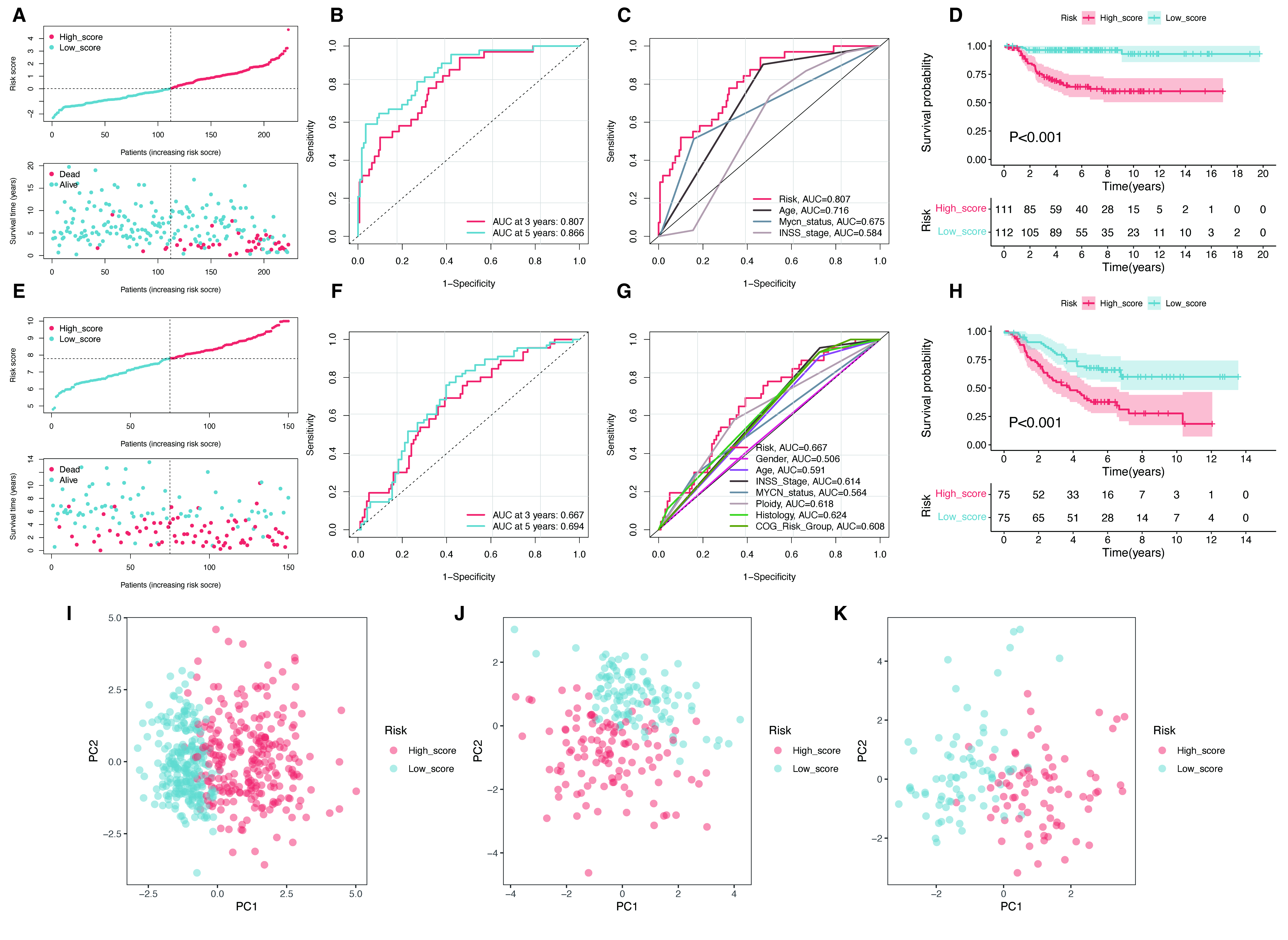

Supplement: Figure S1 — The workflow of the present study. [file DataSheet_1.zip › Supplementary material/FigureS5.tif]

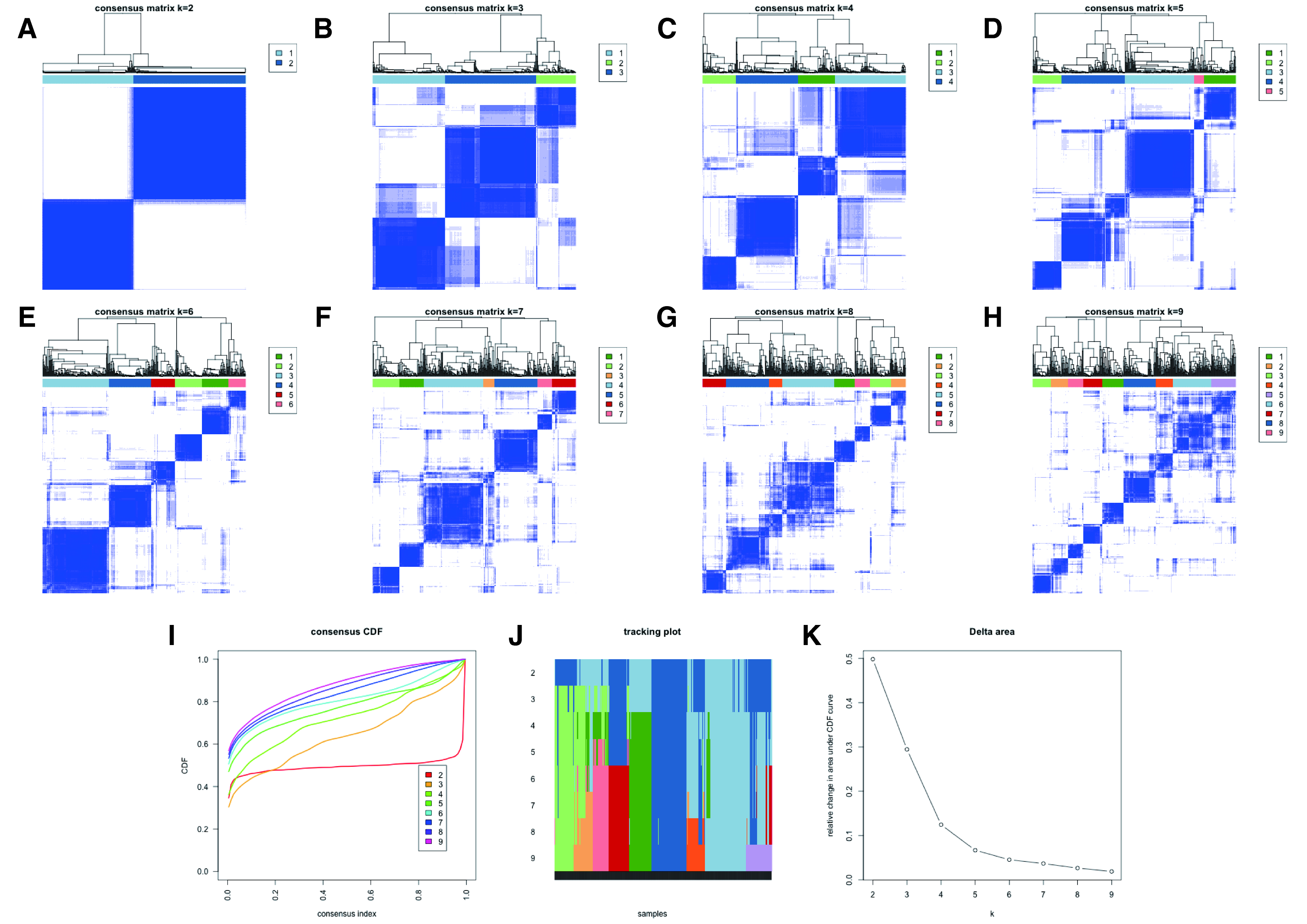

Supplement: Figure S1 — The workflow of the present study. [file DataSheet_1.zip › Supplementary material/FigureS2.tif]

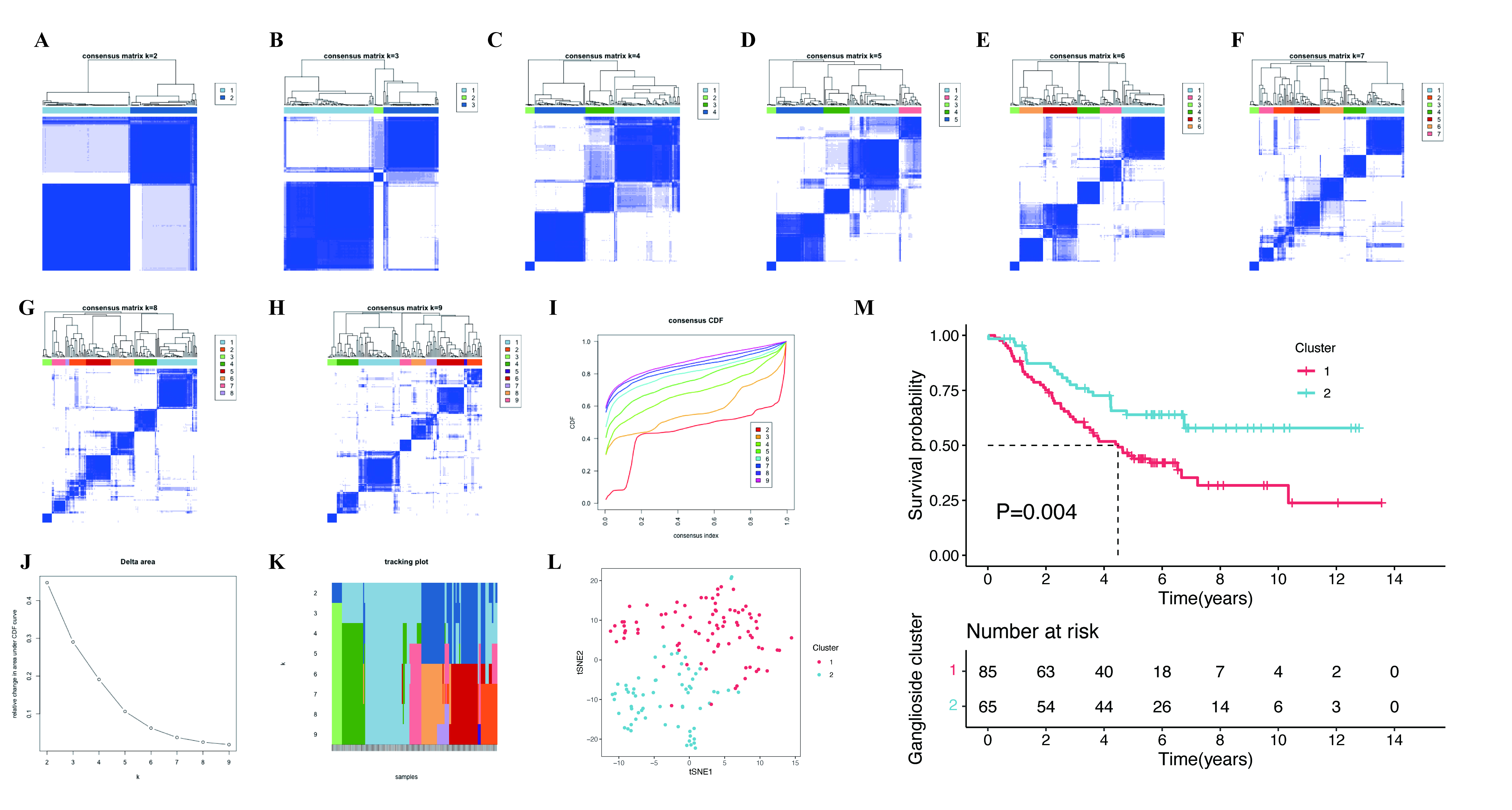

Supplement: Figure S1 — The workflow of the present study. [file DataSheet_1.zip › Supplementary material/FigureS3.tif]
